# The blood transcriptome prior to ovarian cancer diagnosis: A case-control study in the NOWAC postgenome cohort

**DOI:** 10.1371/journal.pone.0256442

**Published:** 2021-08-27

**Authors:** Mie Jareid, Igor Snapkov, Marit Holden, Lill-Tove Rasmussen Busund, Eiliv Lund, Therese Haugdahl Nøst

**Affiliations:** 1 Faculty of Health Sciences, Department of Community Medicine, UiT – The Arctic University of Norway, Tromsø, Norway; 2 Department of Immunology, Oslo University Hospital Rikshospitalet and University of Oslo, Oslo, Norway; 3 Norwegian Computing Center, Oslo, Norway; 4 Faculty of Health Sciences, Department of Medical Biology, UiT – The Arctic University of Norway, Tromsø, Norway; 5 Cancer Registry of Norway, Oslo, Norway; Peter MacCallum Cancer Institute, AUSTRALIA

## Abstract

Epithelial ovarian cancer (EOC) has a 5-year relative survival of 50%, partly because markers of early-stage disease are not available in current clinical diagnostics. The aim of the present study was to investigate whether EOC is associated with transcriptional profiles in blood collected up to 7 years before diagnosis. For this, we used RNA-stabilized whole blood, which contains circulating immune cells, from a sample of EOC cases from the population-based Norwegian Women and Cancer (NOWAC) postgenome cohort. We explored case-control differences in gene expression in all EOC (66 case-control pairs), as well as associations between gene expression and metastatic EOC (56 pairs), serous EOC (45 pairs, 44 of which were metastatic), and interval from blood sample collection to diagnosis (≤3 or >3 years; 34 and 31 pairs, respectively). Lastly, we assessed differential expression of genes associated with EOC in published functional genomics studies that used blood samples collected from newly diagnosed women. After adjustment for multiple testing, this nested case-control study revealed no significant case-control differences in gene expression in all EOC (false discovery rate q>0.96). With the exception of a few probes, the log_2_ fold change values obtained in gene-wise linear models were below ±0.2. P-values were lowest in analyses of metastatic EOC (80% of which were serous EOC). No common transcriptional profile was indicated by interval to diagnosis; when comparing the 100 genes with the lowest p-values in gene-wise tests in samples collected ≤3 and >3 years before EOC diagnosis, no overlap in these genes was observed. Among 86 genes linked to ovarian cancer in previous publications, our data contained expression values for 42, and of these, tests of *LIME1*, *GPR162*, *STAB1*, and *SKAP1*, resulted in unadjusted p<0.05. Although limited by sample size, our findings indicated less variation in blood gene expression between women with similar tumor characteristics.

## Introduction

Epithelial ovarian cancer (EOC) is the eighth most common cancer among Norwegian women, who have a 1.3% risk of developing this cancer by the age of 75 years. Further, age-standardized rates show that EOC is the fifth most common cause of cancer death [1]. EOC is often diagnosed in late stages, with 70% of cases diagnosed with stage III or IV disease. This is partly because markers of early-stage disease are not available in current clinical diagnostics. The symptoms that could lead to EOC diagnosis tend to manifest only after metastasis has already occurred, at which point curative treatment is difficult to achieve. The most common EOC subtype, serous carcinoma, is associated with a particularly poor prognosis [2].

The origin and pathogenesis of EOC vary by subtype, and are still not completely understood. Models have suggested that serous tumors exist as *in-situ* or stage I or II invasive tumors for a median of 5.1 years (95% confidence interval [CI]: 3.2–8.1 years), and advancement to stage III or IV can occur up to 2 years (median 0.8, 95% CI: 0.4–1.9 years) before diagnosis [3].

Ovarian malignancies are associated with skewed proportions of circulating immune cell types, and immunologic studies suggest induction of tumor tolerance through local, and potentially also systemic, immunosuppression mechanisms [4]. Functional genomic studies of circulating immune cells collected at EOC diagnosis have identified markers of risk, presence of tumor in patients, or prognosis [5–13]. However, few have investigated the blood transcriptome [8,10–13].

Whereas blood collected postdiagnostically reflects clinical cancer, random sampling of the general population allows researchers to study persons at different prediagnostic stages of tumorigenesis [14]. The aim of the present study was to investigate whether EOC is associated with transcriptional profiles in blood collected up to 7 years before diagnosis. For this, we used RNA-stabilized whole blood, which contains circulating immune cells, from a sample of EOC cases from the population-based Norwegian Women and Cancer (NOWAC) postgenome cohort. We explored case-control differences in gene expression in all EOC, as well as associations between gene expression and metastatic EOC, serous EOC, and interval from blood sample collection to diagnosis (≤3 or >3 years). Lastly, we assessed differential expression of genes associated with EOC in published functional genomics studies that used blood samples collected from newly diagnosed women.

## Materials and methods

### Study population and sample collection

The present case-control study was nested within the NOWAC postgenome cohort, a subcohort of the NOWAC Study [15]. The NOWAC postgenome cohort is a population-based, prospective study initiated with the purpose of exploring associations between blood gene expression and cancer, with the inclusion of questionnaire information on a variety of exposures and lifestyle factors. Participants were recruited to the NOWAC Study by mail; those who consented to donate blood received a sampling kit with PAXgene blood collection tubes with RNA-preserving buffer (Preanalytix GmbH, Hembrechtikon, Switzerland). Participants then took this kit to a general practitioner’s office, where the blood sample was collected. Between 2003 and 2006, blood samples from close to 50,000 women born between 1943 and 1957 were collected [16] and shipped to the study center, where they were stored at -80 degrees Celsius between 24 hours and 3 days after their collection.

#### Case ascertainment and assignment of matched controls

Epithelial ovarian cancer cases were identified through linkage to the Cancer Registry of Norway using the personal identification number assigned to all Norwegian citizens and permanent residents. Norwegian health care providers are obligated to report all cancer cases to the registry, which ensures near complete national follow-up [17]. Participants of the NOWAC postgenome cohort with registered cancer of the ovary or fallopian tube (International Classification of Diseases revision 7, location 175) diagnosed between April 2004 and April 2011 (n = 95) were eligible for inclusion in this analysis. Tumors were then categorized as borderline, non-epithelial, EOC, and serous EOC; metastasis status was categorized as none, any, or unknown. Controls were matched to cases by birth year and blood sample storage time.

#### Questionnaire variables

On the day of blood sample collection, participants completed a two-page questionnaire concerning recent exposures. Information on variables known to be associated with EOC risk [18] and with gene expression in leukocytes was extracted from this questionnaire, and from NOWAC Study questionnaires: body mass index (BMI) [19], current smoking [20] including number of cigarettes smoked, parity [21], menopausal status [22], and current hormone replacement therapy (HRT) use [23]. We also included current oral contraceptive (OC) use, which modulates EOC risk and could influence gene expression.

### Sample processing

Blood samples were processed at the Genomics Core Facility at the Norwegian University of Science and Technology according to the protocols of kit manufacturers. Samples from case-control pairs were processed together, blinded for case/control status. Total RNA was extracted from whole blood using the PAXgene Blood miRNA Kit (Qiagen GmbH, Hombrechtikon, Switzerland), and cRNA was prepared with the Illumina TotalPrep-96 RNA Amplification Kit (Ambion Inc., Austin, TX, USA). RNA quantity and purity were assessed using a NanoDrop ND 8000 spectrophotometer (ThermoFisher Scientific, Wilmington, DE, USA), and RNA integrity was assessed using Bioanalyzer capillary electrophoresis (Agilent Technologies, Palo Alto, CA, USA). cRNA was hybridized to Illumina HumanHT-12 v4 Expression BeadChip microarrays (Illumina, Inc. San Diego, CA, USA). Illumina GenomeStudio software was used to extract the raw data.

#### Preprocessing of microarray data

Background correction was performed using negative control probes (limma package, nec function) [24]. Probes reported by Illumina to be of poor quality, that were not annotated, that had a detection p-value <0.05, or that were present in less than 70% of the samples, were filtered out. Quantile normalization (lumi, LumiN function) [25] and log_2_ transformation (lumi, LumiT) was performed on the expression values. Finally, probes were mapped and annotated (lumi, nuID2RefSeqID and illuminaHumanv4.db). If multiple probes mapped to the same gene, all were kept in the dataset as duplicates/triplicates.

### Statistical analysis

**Fig 1:**
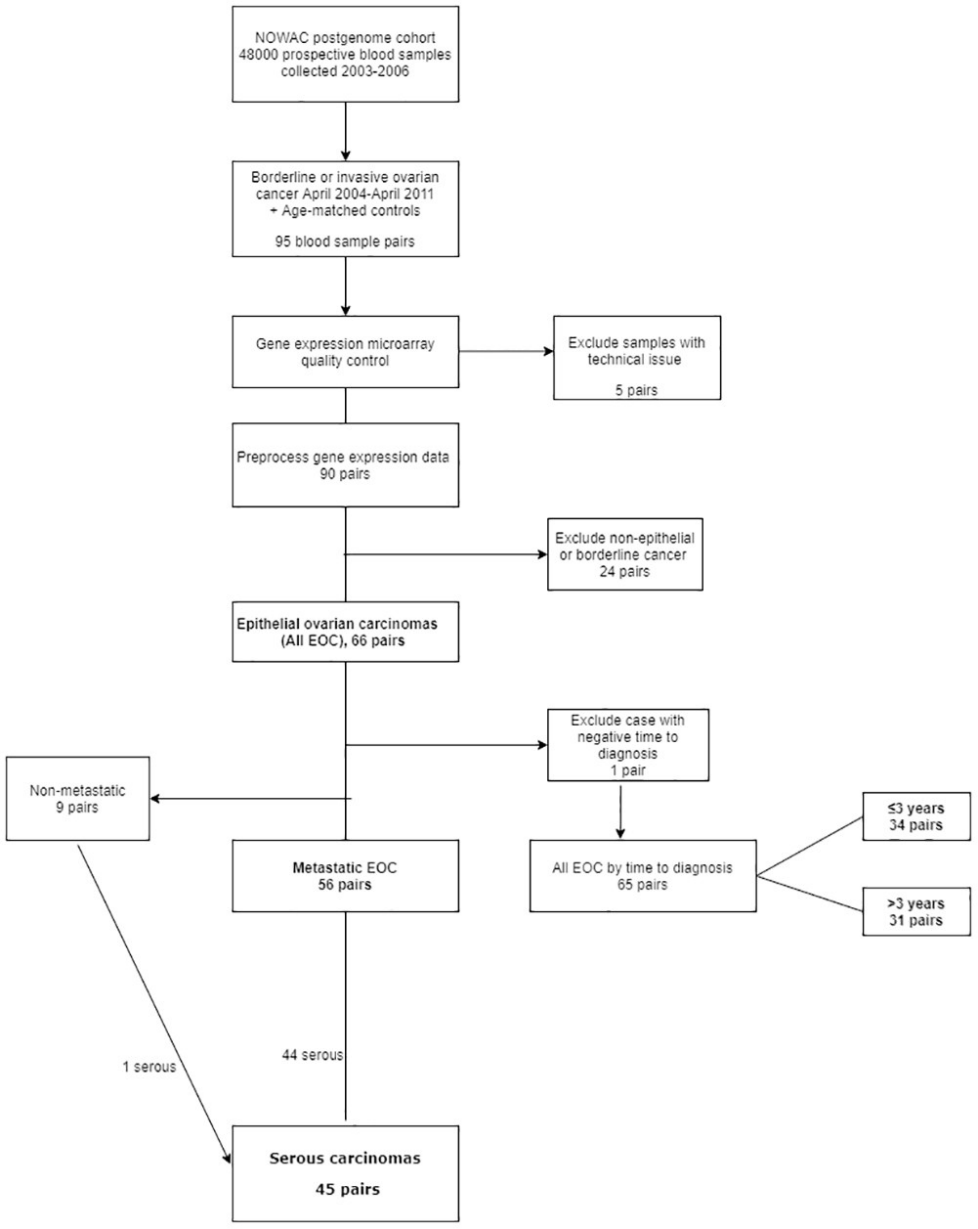
Flow chart of exclusions and analytic groups in gene expression analyses. Bold text indicates analyzed groups. The group “All EOC by time to diagnosis” was used in the analysis of all EOC adjusted for leukocyte populations.

Preliminary quality control of laboratory measurements resulted in the exclusion of five case-control pairs; therefore 90 case-control pairs were included in the preprocessing of microarray data. After preprocessing, the dataset included 12,153 probes for 9,633 genes across 90 cases and 90 controls. We then further excluded cases with borderline tumors (20 pairs) and non-epithelial tumors (4 pairs), leaving 66 case-control pairs in the final dataset. We assessed case-control differences in gene expression in all EOC (66 pairs), as well as associations between gene expression and metastatic EOC (56 pairs), serous EOC (45 pairs, 44 of which were metastatic), and interval from blood sample collection to diagnosis (≤3 years and >3 years, 34 pairs and 31 pairs, respectively). Exclusions and analytical samples are shown in Fig 1. To protect the identity of participants, date of diagnosis was generalized to the month of diagnosis. This resulted in negative follow-up time for one case, and exclusion of this case-control pair from the analysis of blood samples collected ≤3 years before diagnosis. The questionnaire variables BMI (< median 25.8, ≥25.8), current smoking (yes/no), parity (0, 1–2, 3–4, ≥5), menopausal status (pre- or perimenopausal, postmenopausal), current HRT use (yes/no), current OC use (yes/no) were evaluated as potential confounders by testing their association with case status by two-sided t-tests or chi-square tests. Further, to facilitate the evaluation of confounding by differences in leukocyte populations between cases and controls, we estimated leukocyte fractions in individual samples based on gene expression values, using the cell deconvolution procedure Cibersort and the LM22 signature matrix [26]. Variables that were associated with both case/control status (p<0.1) and gene expression (global test [see below], family-wise error-rate adjusted p<0.05) were adjusted for in the analyses.

### Assessment of case-control differences in gene expression

To explore case-control differences in gene expression in all EOC, we computed a dissimilarity matrix with Euclidean distances and applied hierarchal clustering using Ward’s method to create a dendrogram. Further, we made a multidimensional scaling plot to display distances between samples. We used the global test [27] to assess case/control sample status as a function of difference in overall gene expression in all EOC, metastatic EOC, and serous EOC. Using linear models in the limma package [24], we assessed differences in expression of single genes (log_2_ fold change [FC] values) between cases and matched controls in all EOC, metastatic EOC, serous EOC, and EOC cases diagnosed ≤3 years and >3 years after blood sample collection.

We used the global test [27] to evaluate associations between potential confounding variables and gene expression overall, and created an adjusted gene-wise model of all EOC. To explore expression differences in sets of genes, we used the mroast method (using 10^5^ rotations) [28] to test gene sets from the C2, C5, C7 [29,30] and KEGG [31] collections in the Broad Institute databases [32].

Genes were considered differentially expressed if the false discovery rate (FDR)-adjusted p-value (q value) was <0.05. We present non-FDR-adjusted p-values in the tables and text. The open source softwares R [3.1.2 and 3.2.1] (Vienna, Austria; www.r-project.org) and Bioconductor (bioconductor.org) were used for the analyses, with the exception of the chi-square test [33].

#### Gene Ontology enrichment

To explore the biological functions of the genes indicated according to case-control differences in expression, we used the R package clusterProfiler v.3.12.0 [34], which assesses potential overrepresentation of Gene Ontology (GO) terms [35,36]. We assessed the 100 probes with the lowest p-values in the limma models without covariate adjustments.

#### Differential expression of genes identified in published functional genomics studies

We used the metastatic EOC group to test case-control differences in the expression of seven sets of 5–33 genes reported to be associated with EOC in published functional genomics studies that used blood samples collected from newly diagnosed women. Of these, two gene sets were identified in whole blood gene expression studies comparing patients given a poor or better prognosis according to tumor characteristics [10,11]. Five gene sets were identified in case-control studies of DNA methylation in circulating leukocytes. We tested for differential expression of genes adjacent to CpG sites where differential methylation was reported indicative of EOC case status [6,9], CpGs indicative of EOC predisposition [5]; CpGs where methylation mediates genetic risk of EOC [7], and a set of genes where expression levels was suggested to mediate genotype-associated risk of EOC [8]. We tested a total 86 genes using a two-sided t-test for each gene, and did not adjust the p-values for multiple testing.

### Ethics

The Regional Committee for Medical and Health Research Ethics (REC North) approved the NOWAC Study, the storage of blood samples, and the gene expression analyses in the present study. The Norwegian Data Inspectorate approved the linkages to the Cancer Registry of Norway. Participants received written information about the study and their right to withdraw. Signing the informed consent form, or completing a written questionnaire and donating a blood sample, was regarded as informed consent for cohort enrollment.

## Results

### Participant characteristics

Mean age at blood sample collection among cases and controls was 56.5 years; mean age at EOC diagnosis among cases was 59.3 years. Cases and controls did not differ significantly with regard to questionnaire variables. Both cases and controls tended toward being overweight, with a mean BMI of approximately 27, and 23% were current smokers. Fewer cases than controls were nullipara, and more cases than controls had 3–4 children (32% vs 24%), but parity distribution did not differ overall (p = 0.78). In both groups, approximately 90% were postmenopausal, 20% were current HRT users, and there were no current OC users (Table 1).

**Table 1:** Participant characteristics on day of blood sample collection.

Variable	Cases (n = 66)	Controls (n = 66)	P-value^a^
	Mean (SD) or frequency (%)		
**Age (years)**	56.5 (3.7)	56.5 (3.7)	-
**Time to epithelial ovarian cancer diagnosis (years)**	2.8	-	
**Body mass index (kg/m^2^)**	26.8 (6.5)	27.0 (4.5)	0.81
**Current smoker**	15 (23%)	15 (23%)	-
Number of cigarettes yesterday	11.8 (7.9)	10.1 (8.1)	0.57
Number of cigarettes today	1.9	2.9	0.25
**Parity**			0.78^b^
0	6 (9%)	7 (11%)	
1–2	37 (56%)	40 (60%)	
3–4	21 (32%)	16 (24%)	
≥5	2 (3%)	3 (5%)	
**Menopausal status**			
Pre- or perimenopausal	6 (9%)	9 (14%)	-
Postmenopausal	59 (91%)	56 (86%)	0.44
**Current hormone replacement therapy use (%)**	13 (20%)	14 (21%)	0.47
**Current oral contraceptive use** ^c^	0	0	

^a^ p-values obtained from a two-sided t-test.

^b^ p-value comparing the distribution of number of children among cases and controls was obtained from a chi-square test.

^c^38% missing values.

Of the 66 women with EOC, 56 (85%) had any metastasis, nine had no metastasis, and one had unknown metastasis status (Table 2). EOC subtype distribution included endometrioid (6%), clear cell (6%), mucinous (4.5%), other/non-specified histologies (15%), and serous EOC (n = 45), which constituted 68% of all EOC (24 with blood sample collected ≤3 years and 21 collected >3 years before diagnosis) (Table 2). Among serous EOC, the percentage of high, low, and unknown grade was 69%, 4%, and 27%, respectively. Of the high-grade serous EOC, 40% and 60% had blood samples collected ≤3 years and >3 years before diagnosis, respectively. Among those with low- and unknown-grade serous EOC, the corresponding distributions were 50% and 50%, and 60% and 40%, respectively. Compared to controls, cases had larger estimated mean fractions of CD8+ T cells and plasma cells (p = 0.08 and p = 0.07, respectively), and smaller fractions of neutrophils, monocytes, and resting mast cells (p = 0.06, p = 0.08, and p = 0.02, respectively; S1 Table).

**Table 2:** Distribution of epithelial ovarian cancer (EOC) cases in analytical groups (bold text) of case-control differences in gene expression.

Interval from blood sample collection to diagnosis	≤3 years	>3 Years	Sum
**All EOC**	**35** ^a^	**31**	**66**
**Metastatic EOC**	30	26	**56**
Non-metastatic EOC	5	5	10^b^
**Serous EOC**	24	21	**45**
Non-serous EOC	11	10	21

^a^One case was diagnosed same month as sample collection and this case-control pair was excluded in the single-gene linear models of samples collected ≤3 years before diagnosis.

^b^One case with unknown metastasis status was categorized as non-metastatic.

### Case-control differences in gene expression

Hierarchal clustering of all EOC cases and controls (S1 Fig) and multidimensional scaling of pairwise distances between case-control pairs (S2 Fig) showed no tendency toward clustering of samples by case/control status. The global tests of all EOC, metastatic EOC, and serous EOC resulted in p-values of 0.87, 0.72, and 0.67, respectively. The single-gene linear models did not identify any genes differentially expressed between cases and controls (FDR q-values ranged from 0.96–0.99; S2–S6 Tables). The lowest p-value was observed in metastatic EOC (*FBLN5*; log_2_FC = 0.07, p = 0.0002) (S3 Table). In all EOC, the lowest p-value was observed for the probe *ENSA* (log_2_FC 0.06, p = 0.01) (S2 Table). The gene set analyses did not indicate any differentially expressed set of genes (lowest unadjusted p-value = 0.001).

**Fig 2:**
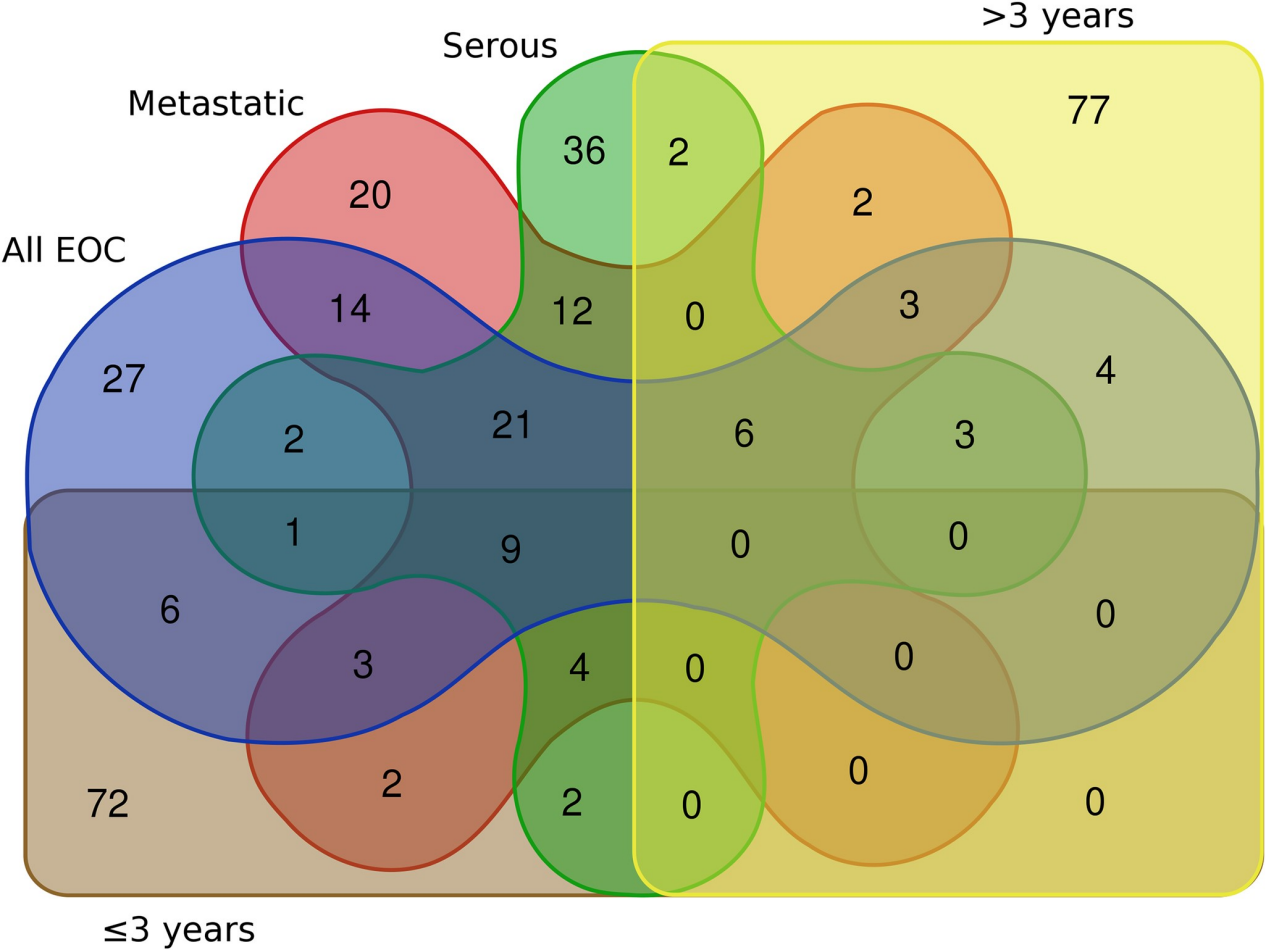
Overlap between the 100 probes with lowest p-values in single-gene linear models (case-control) of prospective blood samples from all cases of epithelial ovarian cancer (EOC; 66 pairs) and subgroups (metastatic at diagnosis (56 pairs), serous subtype (45 pairs), or interval to diagnosis (≤3 years or >3 years; 34 and 31 pairs, respectively)). The 100 probes are listed in S2–S6 Tables. (Created with BGE Venn diagram tool, Ghent University).

S2–S6 Tables list the 100 probes with lowest unadjusted p-values in single-gene linear models of all EOC and investigated subgroups (Fig 1). We observed 36 overlapping probes in all EOC, metastatic EOC, and serous EOC (Fig 2). However, when separated into groups of blood samples collected ≤3 years and >3 years before diagnosis, the lists of probes with the 100 lowest p-values did not overlap (Fig 2).

Among the 100 probes with the lowest p-values, few log_2_FC values exceeded ±0.2 (Table 3; S2 Fig shows the volcano plot for all EOC). The largest absolute log_2_FC values observed were for *DEFA1B* (log_2_FC = 0.64, p = 0.01) in blood samples collected ≤3 years before diagnosis, and *LOC644936* (log_2_FC = -0.53, p = 0.02) in serous EOC. These probes did not occur among the 100 lowest p-values in any other group.

**Table 3:** Probes with the 20 greatest absolute log_2_FC values^a^ among the 100^b^ lowest p-values in single-gene linear models (case-control) of prospective blood samples from all cases of epithelial ovarian cancer (EOC) and subgroup analyses by clinicopathologic characteristics and interval to diagnosis.

All EOC N = 66	log_2_FC	Metastatic EOC n = 56	log_2_FC	Serous EOC n = 45	log_2_FC	≤3 years n = 34	log_2_FC	>3 years n = 31	log_2_FC
**Positive log**_**2**_ **FC**									
*GZMH*	0.31	***LOC642161***	0.16	*BTN3A2*	*0*.*20*	*DEFA1B*	*0*.*64*	*LEF1*	*0*.*21*
*SNHG5*	0.25	*CD2*	0.15	***LOC642161***	*0*.*19*	*C21orf7*	*0*.*25*	*ETS1*	*0*.*20*
***MIAT***	0.15	*EEF1G*	0.13	*CD7*	*0*.*16*	*DEFA4*	*0*.*23*	*EEF1G*	*0*.*17*
***LOC642161***	0.14	*GIMAP5*	0.13	*GIMAP5*	*0*.*16*	***MIAT***	*0*.*20*	*GLO1*	*0*.*16*
***CD8A***	0.12	*CD3E*	0.13	***RPL8***	*0*.*13*	*MCOLN2*	*0*.*19*	*EEF1A1*	*0*.*14*
***RPL8***	0.11	***RPL8***	0.12	***LOC387882***	*0*.*10*	*ASCL2*	*0*.*16*	*EDG1*	*0*.*13*
*LOC728855*	0.11	***CD8A***	0.12	*KLHDC4*	*0*.*10*	*DGKQ*	*0*.*15*	*NUP88*	*0*.*12*
***APOBEC3G***	0.09	***APOBEC3G***	0.11	*TSEN54*	*0*.*09*	***LOC642161***	*0*.*15*	*C10orf32*	*0*.*11*
***LOC387882***	0.08	*CPT1B*	0.10	*HERC1*	*0*.*09*	*MT1X*	*0*.*13*	*CCT8*	*0*.*11*
*RAB11FIP5*	0.08	*NELF*	0.09	*SAMD3*	*0*.*09*	*MT1E*	*0*.*13*	*EXOSC8*	*0*.*11*
**Negative log**_**2**_ **FC**									
***PPT1***	-0.13	*RHOQ*	-0.15	***TMEM154***	-0.19	*C20orf111*	-0.14	*SCAP*	-0.17
***NA* (*AL080095*)**	-0.13	***NA (AL080095*)**	-0.15	***TAOK1***	-0.19	*LAT2*	-0.14	*TRPC4AP*	-0.18
***MPPE1***	-0.13	***FLJ22662***	-0.15	*FLJ22662*	-0.20	*TRIB1*	-0.15	*ELANE*	-0.19
***TMEM154***	-0.13	***MPPE1***	-0.17	*IGSF6*	-0.20	*OSBPL8*	-0.15	*ANXA11*	-0.19
***TAOK1***	-0.13	***TAOK1***	-0.18	*RYBP*	-0.21	*RRP7A*	-0.15	*VCL*	-0.19
***USF1***	-0.14	***TMEM154***	-0.18	***CD93***	-0.23	*CYBRD1*	-0.16	*TSPAN9*	-0.21
*LAIR2*	-0.16	*TMEM154-dupl*	-0.18	*FCGR3B*	-0.25	*CXCR5*	-0.17	***CD93***	-0.22
*LAIR2-dupl*	-0.17	***CD93***	-0.20	***KCTD12***	-0.25	*FLJ22662*	-0.18	***TAOK1***	-0.22
***CD93***	-0.17	*FCGR3B*	-0.21	*PI3*	-0.40	*SKAP2*	-0.18	***USF1***	-0.25
***KCTD12***	-0.20	***KCTD12***	-0.24	*LOC644936*	-0.53	***PPT1***	-0.21	*GP9*	-0.38

Bold text indicates probes that occurred among the 10 probes for all EOC as well as another group.

^a^Unadjusted p-values for the displayed probes ranged from 0.001 to 0.03, and were lowest in metastatic EOC.

^b^The 100 probes with lowest p-values are listed by p-value in S2–S6 Tables.

No questionnaire variables were significantly associated with case-control status (Table 1) or with gene expression overall (p>0.12). The estimated leukocyte fractions found to be associated with case-control status (neutrophils, CD8+ T cells, monocytes, resting mast cells, and plasma cells; S1 Table) were associated with gene expression overall (p = 0.02, 0.04, 1.75e-11, 3.00e-05, 5.00e-06, respectively). Therefore, the adjusted gene expression model included these five leukocyte types and no questionnaire variables. The lists of 100 probes with lowest p-values resulting from the unadjusted and adjusted models of all EOC (S2 and S7 Tables) overlapped by 29 probes.

#### Gene Ontology enrichment

**Fig 3:**
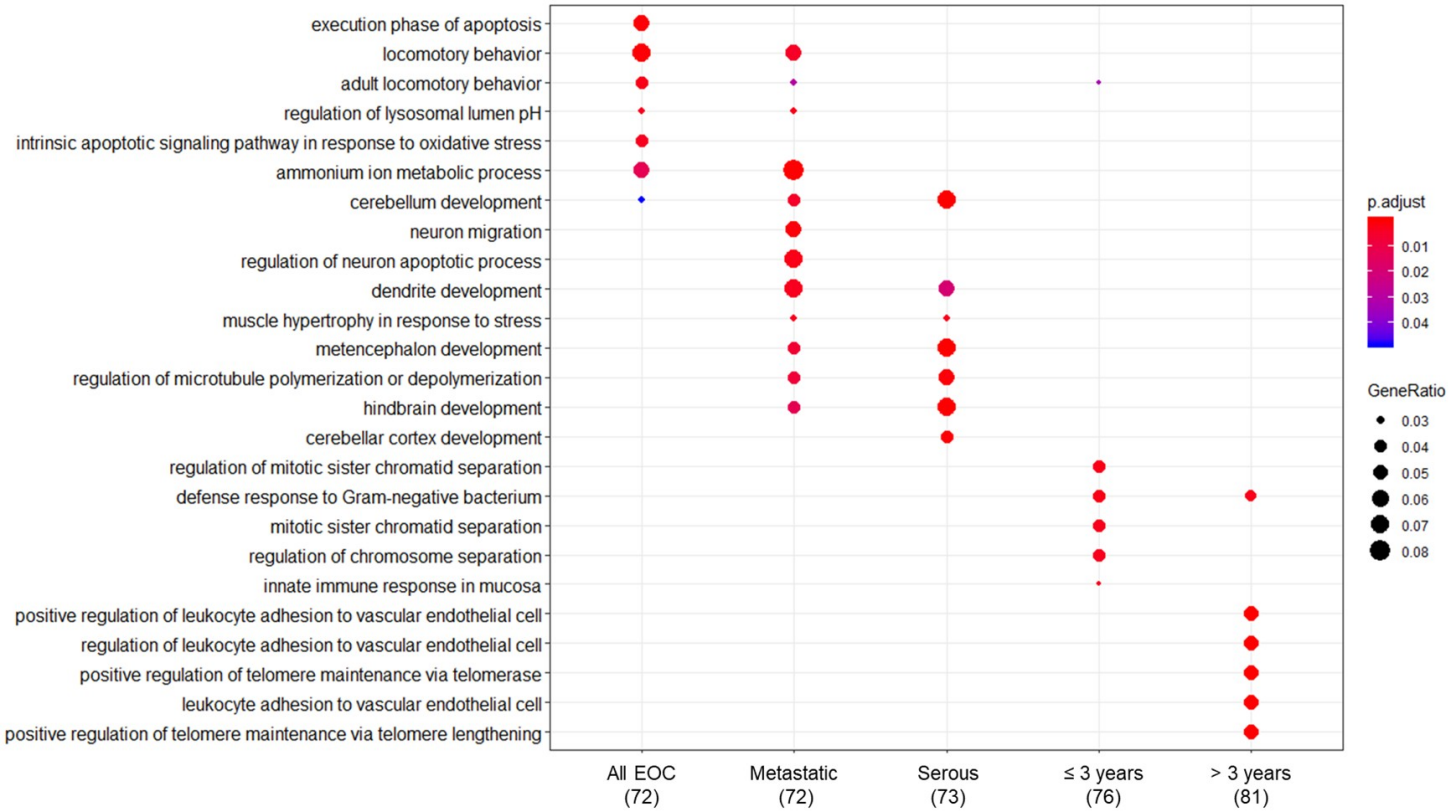
Gene Ontology (GO) enrichment of biological processes among the 100 probes with the lowest p-values in single-gene linear models (case-control) of blood samples from all cases of epithelial ovarian cancer (66 pairs) and according to metastasis status (56 pairs), serous subtype (45 pairs), and interval to diagnosis (≤3 years or >3 years; 34 and 31 pairs, respectively). Numbers below each column indicate the number of probes for which GO categories could be found. The figure presents the five GO categories with lowest p-values. Enriched GO categories (p<0.05) beyond the top five are included in addition if they are among the five most enriched of one of the other investigated groups. S8 Table presents the complete GO enrichment list.

S8 Table displays GO categories related to biological processes overrepresented among the 100 probes with the lowest p-values in all EOC, metastatic EOC, serous EOC, and in blood samples collected ≤3 years or >3 years before diagnosis. Fig 3 presents the GO categories with the lowest p-values in each group, as well as GO categories that overlapped between the groups. In all EOC, the main enriched categories were “execution phase of apoptosis” and "intrinsic apoptotic signaling pathway in response to oxidative stress” (contributing genes: *TAOK1*, *STK24*, *RFFL*, *HTRA2*, *DIABLO*), “locomotory behavior” (*CLN6*, *NR4A2*, *PPT1*, *PDE1B*, *HTRA2*), and “regulation of lysosomal lumen pH” (*CLN6*, *PPT1*). With the exception of *PDE1B*, these probes displayed negative log_2_FC values.

In metastatic EOC, the main enriched categories were “ammonium ion metabolic process” (*NR4A2*, *PLA2G7*, *PDE1B*, *PLBD1*, *CHKA*, *CPT1B*), “neuron migration” and “regulation of neuron apoptotic process” (*NR4A2*, *MEF2C*, *PPT1*, *CDK5R1*, *NSMF*), and “dendrite development” (*EZH2*, *MEF2C*, *CDK5R1*, *NSMF*, *CD3E*). Among these transcripts, *PDE1B*, *CD3E*, *EZH2*, and *NSMF* displayed positive log_2_ FC values.

In serous EOC, enriched GO categories were “cerebellum development”, “metencephalon development”, “hindbrain development”, “cerebellar cortex development” (*CDK5R1*, *PAK1*, *EZH2*, *SERPINE2*, *HERC1*), and “regulation of microtubule polymerization or depolymerization” (*CDK5R1*, *PAK1*, *FES*, *TAOK1*). The four genes associated with microtubule polymerization displayed negative log_2_FC values, while the remaining log_2_FC values were positive. In blood samples collected ≤3 years before diagnosis, the main enriched GO categories were “regulation of mitotic sister chromatid separation” (*PTTG3P*, *CENPE*, *PTTG1*), “defense response to Gram-negative bacterium” (*DEFA1B*, *DEFA4*, *TNFRSF14*), and “innate immune response in mucosa” (*DEFA1B*, *DEFA4*). These probes displayed positive log_2_FC values, and none of the genes were included in the top five enriched categories of the other groups.

Finally, in samples collected >3 years before diagnosis, the enriched GO categories were “positive regulation of leukocyte adhesion to vascular endothelial cell” (*NFAT5*, *ICAM1*, *ELANE*, *ETS1*) and “positive regulation of telomere maintenance via telomerase and telomere lengthening” (*CCT2*, *CCT8*, *MAPKAPK5*, *HMBOX1*). In the first mentioned GO category, all probes except *ETS1* displayed negative log_2_FC values, whereas in the latter, all except *HMBOX1* were positive.

#### Differential expression of genes identified in published functional genomics studies

Our metastatic EOC group contained expression values for 42 of the 86 genes from relevant publications. S9 Table lists the genes, the log_2_FC values we observed for these probes, and the difference in expression or methylation status in the original studies. From the two gene sets obtained from whole blood gene expression studies, our data contained expression values for two of six genes identified by qPCR [10] and five of six genes previously identified using gene expression microarrays [11]. The lowest p-values we observed from these gene sets were for the probes *CTNNA1* (log_2_FC = -0.05, p = 0.09) and *NCALD* (log_2_FC = 0.08, p = 0.08).

Our dataset contained expression values for genes adjacent to more than two-thirds of the methylation sites identified by Teschendorff et al. [5] and Koestler et al. [7]. Among these gene sets, three probes had p-values <0.05: *LIME1* (log_2_FC = 0.11, p = 0.05) and *GPR162* (log_2_FC = -0.17, p = 0.04) from Teschendorff et al. [5], and *STAB1* (log_2_FC = -0.05, p = 0.01) from Koestler et al. [7]. Our dataset contained expression values for less than half of the genes adjacent to methylation sites identified by Fridley et al. [6], Li et al. [9], and Yang et al. [8]. Among these, the test of *SKAP1* identified by Yang et al. [8] resulted in a p-value <0.05 (log_2_FC = 0.07, p = 0.04).

## Discussion

This nested case-control study of gene expression in whole blood collected up to 7 years prior to EOC diagnosis revealed no statistically significant global or gene-wise associations with EOC case status. The data were high-dimensional, which hampered the statistical power, and the sample size limited the possibilities for analyses according to tumor characteristics or time intervals. Nevertheless, group differences in p-values indicated smaller variation in analyses restricted to metastatic EOC or serous EOC, and greater variation in blood samples collected ≤3 years before diagnosis. Compared to controls, cases had larger estimated mean fractions of CD8+ T cells and plasma cells and smaller fractions of neutrophils, monocytes, and resting mast cells. Adjusting for these differences altered the ranking of probes by p-value, but otherwise did not change the results. In targeted gene-wise tests of 42 genes associated with EOC in previous genetic, epigenetic, and transcriptomic studies in blood, four genes were nominally significant among the metastatic cases in the present study.

### Case-control differences in gene expression

Neither unsupervised clustering methods, the global test, single-gene linear models, nor gene sets identified statistically significant case-control differences in blood gene expression. With the exception of a few probes, the log_2_FC values obtained in gene-wise linear models were less than ±0.2. A log_2_FC value of 0.2 equals a fold change of 1.15, which, if interpreted as an indicator of effect size in epidemiological terms, corresponds to a 15% increase in risk of disease.

As no genes were significantly differentially expressed in this study, the interpretation of single genes was kept to a minimum. The probe with lowest p-value in all EOC, *ENSA*, was also among the 100 probes with the lowest p-values in metastatic EOC and serous EOC, and displayed a larger log_2_FC value in blood samples collected >3 years before diagnosis. *ENSA* encodes α-endosulfine, a cytoplasmic unstructured phosphoprotein with various binding partners depending on cellular context, and regulatory functions depending on its phosphorylation state [37]. Its functions include regulation of cell cycle and platelet activity [38]. In relation to EOC, a small study of serum autoantibodies detected in women with EOC has indicated ENSA as a potential autoantigen [39].

Among the probes with highest log_2_FC values in all EOC were four genes (*GZMH*, *APOBEC3G*, *SNHG5*, *MIAT*) that have previously been indicated in studies targeting EOC. In a network analysis of serum proteins, EOC case status was associated with levels of granzyme H (GZMH) in blood samples collected >34.5 months prior to diagnosis [40]. A study of tumor transcriptome data associated quantities of the long, non-coding RNAs *SNHG5* and *MIAT* with EOC stage [41], while *APOBEC3G* expression in tumor infiltrating lymphocytes has been associated with EOC survival [42,43]. These transcripts could potentially be of interest in future studies of circulating markers of EOC, but could not be considered as associated with EOC in our whole transcriptome analysis.

#### Case-control differences by metastasis status, histological subtype, and interval to diagnosis

The majority of the cases in this study were metastatic at diagnosis, and the majority of the metastatic cases were of serous subtype. The lower p-values in these subgroup analyses compared to all EOC indicated less variation in gene expression between blood samples from women with similar tumor characteristics. Previous studies in the NOWAC postgenome cohort that investigated prospective blood samples from women diagnosed with breast cancer [44] and lung cancer [45] found significant case-control differences in gene expression when analyses were restricted to metastatic cancers. It is uncertain whether the lower p-values we observed for metastatic EOC compared to all EOC reflects a similar phenomenon that would have reached statistical significance with a larger sample size.

Our study was based on blood and would detect signals of cancer developing in the ovaries only by association with the composition of the blood transcriptome. Since serous EOC in particular tends to spread while at a low volume [3], early changes in peripheral immune cells could potentially be a more sensitive systemic indicator of malignant disease than substances of tumor origin, which are produced in proportion to tumor mass [9].

The interval from blood sampling to diagnosis in the present study covers the estimated duration of the development of serous EOC from *in-situ* to stage IV metastatic disease [3]. Inferring from the estimations of Brown and Palmer [3], the women in our study who were diagnosed with serous EOC and had blood samples collected ≤3 years before their diagnosis likely suffered from some degree of metastasis at the time of sample collection. Assuming a rapid development of the tumor in the final year before diagnosis [3], the higher p-values and larger log_2_FC values we observed in samples collected ≤3 years before diagnosis could reflect larger transcriptional variation in this group, possibly as an indicator of disease-associated transcriptional dysregulation. The percentage of probes with positive log_2_FC values was 70% in this group, compared to 50% in other groups except for all EOC adjusted for leukocyte populations, where this percentage was also 70%. This could suggest a general upregulation of gene transcription in samples collected ≤3 years before diagnosis, rather than a specific composition of leukocyte types.

In the samples collected >3 years before diagnosis, which could theoretically contain signals of stage I and II serous EOC [3], the case-control differences in gene expression were not as strong. When comparing the 100 probes with the lowest p-values in samples collected ≤3 years and >3 years before diagnosis, no overlap in probes was observed. These groups were similar with regard to the distribution of metastatic and serous EOC. Thus, we observed no common transcriptional profile associated with EOC across the postulated time frame for its development. A recent study used mouse models to confirm shifts in systemic immune status during cancer development [46], and it is possible that if our analyses were designed to capture the dynamics of the disease course, we would have been able to identify similar changes associated with EOC. However, due to the small number of samples, we chose not to perform analyses of shorter time intervals.

#### Gene Ontology enrichment

To explore whether metastasis status, EOC subtype, or time to diagnosis were reflected in biological processes in blood, we compared overrepresented GO categories from the 100 probes with the lowest p-values in single-gene linear models. The overlap of gene lists and shared GO categories (Figs 1 and 2) reflected that all EOC, metastatic EOC, and serous EOC were nested and largely contained the same samples, and that samples collected ≤3 years and >3 years before diagnosis simply represent subdivisions of all EOC.

Among the GO categories indicated in all, metastatic, or serous EOC, locomotory behavior, neuronal migration and central nervous system development have been designated as relevant for the immune system [47]. Migration is a feature of developing neural cells that immune cells share [48]. Overlapping functions of these genes in the immune and neural systems also include the cellular apparatuses related to signaling pathways and cell-to-cell communication [49,50]. Microtubule polymerization and depolymerization, which was enriched in serous EOC, is intrinsic to lymphocyte migration, but also to formation of the immunological synapses necessary for activation of T and B cells [51]. Thus, the main common feature of the overrepresented GO categories for all EOC, metastatic EOC, and serous EOC was their relation to locomotion. If this observation is related to case status, it could suggest that leukocyte migration is affected by EOC.

For blood samples collected ≤3 years before diagnosis, “innate immune response in mucosa” and “defense response to Gram-negative bacterium” were among the main enriched GO categories. Interestingly,”defense response to Gram-negative bacterium" was also overrepresented in blood samples collected >3 years before diagnosis, though neither samples nor probes overlapped. If linked to EOC, the log_2_FC values were suggestive of initial downregulation of this process, followed by upregulation closer to diagnosis.

In blood samples collected >3 years before diagnosis, the categories “positive regulation of leukocyte adhesion to vascular endothelial cell” and “positive regulation of telomere maintenance via telomerase and telomere lengthening” were overrepresented. Telomere maintenance is activated during proliferation of activated T and B cells [52]. While this observation is epidemiologically relevant [52], it could be related to the larger proportion of CD8+ T cells in cases overall. Adhesion to endothelial cells is a core mechanism of leukocyte migration, which adds to the above mentioned results for metastatic and serous EOC.

The RNA species investigated in this study included mRNA and polyadenylated long non-coding RNA, and comprised the transcriptome of all circulating immune cells as well as circulating extracellular RNA. Whole blood transcriptomics may thus offer insight into systemic disease processes or enable discovery of circulating markers of disease. Our study design and sample collection were aimed at performing such explorative analyses; however, our study sample was small, and small differences in expression between cases and controls resulted in gene lists that likely included noise. It has been emphasized that GO databases include certain genes that are annotated to many categories [53] and represent current knowledge of genes. Therefore, we have interpreted GO categories with caution.

#### Estimated leukocyte fractions

The estimated relative sizes of leukocyte populations varied considerably between individuals. On a 10% significance level, EOC cases had slightly larger fractions of CD8+ T cells and plasma cells (adaptive immune system), and smaller fractions of neutrophils, monocytes, and resting mast cells (innate immune system) compared to controls. Adjusting our gene expression models for these leukocyte proportions altered the probes with the lowest p-values, indicating that genes with expression differences according to case-control status were due to differences in these populations.

EOC has been associated with altered proportions of CD8+ T cells, monocytes, and granulocytes (neutrophils, eosinophils, basophils) at diagnosis [7,54], but these studies reported case-control differences opposite to our estimates. Our non-significant observation of higher proportions of regulatory T cells and M2 macrophages in cases (S1 Table) is more in line with previous studies (summarized in [4]). It is possible that our mean estimates conceal a time-dependent shift during the prediagnostic interval, or that we did not estimate the cell types most relevant for EOC [55].

We estimated relative proportions of 22 leukocyte types. The estimates diverged from the normal physiological range [56] in a manner similar to a divergence observed in other recent studies in the NOWAC postgenome cohort [45,57], which indicates bias. The source might be the deconvolution matrix [58] or upstream laboratory or data processing.

#### Differential expression of genes identified in published functional genomics studies

Finally, we used the metastatic EOC group to assess signatures from previous studies of postdiagnostic blood samples from women with EOC. These genes of interest were identified in gene expression studies of patients grouped by tumor characteristics [10,11], or DNA methylation studies of EOC cases and controls [5–9]. Although study designs differed, we could assess how these genes associate with EOC on the transcriptional level in prediagnostic samples. Targeted analyses also let us overcome the problem of multiple testing that arises in explorative analyses.

Gene-wise tests of 42 genes resulted in four probes (*LIME1*, *GPR162*, *STAB1*, *SKAP1*) with p-values <0.05 (S9 Table). We observed the largest log_2_FC values for *LIME1* and *GPR162* from the study by Teschendorff et al. [5]. *LIME1* (Lck interacting transmembrane adaptor 1; log_2_FC = 0.11) is expressed in T cells and B cells, where it links T and B cell receptors to downstream signaling pathways via kinases in the Src family [59]. *GPR162* (G Protein-Coupled Receptor 162; log_2_FC = -0.17) encodes an orphan receptor with adrenaline and noradrenaline as putative ligands [60]. Its mRNA is enriched in neutrophils, monocytes and fallopian tube, but the protein is primarily expressed in the brain [59]. Teschendorff et al. [5] partially attributed the methylation differences they observed to tumor-associated changes in circulating leukocyte composition, and they reported hypermethylation of *LIME1* and *GPR162* in EOC cases. We observed divergent log_2_FC values for these probes, which, considering the cell type specificity of the transcripts, was in line with our estimated differences in leukocyte populations. However, if the expression difference we observed for *GPR162* is partially attributable to a global change in methylation, this could suggest an altered reception of adrenergic signaling [61–63].

*STAB1* (Stabilin 1; log_2_FC = -0.05) from the study of Koestler et al. [7] encodes a scavenger receptor suggested to mark immunosuppressive monocytes and macrophages, where decreased expression appears to increase T cell antitumor cytotoxicity [64]. *SKAP1* (Src kinase-associated phosphoprotein 1; log_2_FC = 0.07) from the gene set of Yang et al. [8] encodes a T cell receptor adaptor protein and is a known EOC risk locus with a possible cell-autonomous role in EOC tumorigenesis [65]. Yang et al. [8] reported two methylation sites for this gene in leukocytes: one site was associated with higher *SKAP1* expression and higher EOC risk, and the other with lower *SKAP1* expression and lower EOC risk. Our observation supports a positive association between EOC and levels of *SKAP1* transcripts in blood, though this could simply reflect the proportion of T cells in our study.

In summary, the genes with nominally significant differential expression coded for receptor proteins and for adaptor proteins involved in Src pathways. These genes derived from methylation signatures of EOC predisposition or early disease [5] and methylation-mediated genetic risk [7,8].

### Strengths and weaknesses

The main weakness of this study is its sample size, which hampered the power of the statistical analyses and limited the methodological possibilities for modeling continuous relationships between gene expression and time to diagnosis. We excluded borderline epithelial tumors *a priori*, which further reduced the sample size. These tumors could have been included as non-metastatic EOC, but they represent a pathological entity separate from invasive carcinomas. We did not evaluate potential confounding by past exposure to exogenous hormones. Further, the NOWAC postgenome cohort has not contributed repeat blood samples at different time points during follow-up, a practice which has proven useful in linking proteomic data to EOC [40]. The present study was designed to be explorative and descriptive. Even though any findings might have been useful for biomarker development, the sample size in this study was insufficient to adopt a training, validation and test approach. There were no clear candidate transcripts to pursue in further analyses as potential biomarkers.

Strengths of this study include an epidemiological design aimed at avoiding sampling bias, and blood sample collection during a period that addresses the need for data on circulating molecular markers from women with early-stage EOC. Further, the case-control pairs were matched on age and sample storage time, and we evaluated potential confounding by leukocyte proportions and risk factors.

We chose an analytical approach commonly used in gene expression studies, and which was in line with another whole blood gene expression study related to EOC [11]. The small case-control differences implies that potential signals in the data are subtle against a noisy background; the data are high-dimensional and the results non-significant when adjusted for multiple testing.

### Conclusion

This nested case-control study did not reveal statistically significant differences in the peripheral blood transcriptome prior to a diagnosis of EOC. The exploration of transcriptional profiles in blood indicated case-control differences that were small in magnitude and did not reach statistical significance when adjusted for multiple testing. The estimated leukocyte population distributions suggested larger proportions of adaptive immune cell types and smaller proportions of innate immune cell types in cases than in controls, and the functional enrichment suggested lower expression of genes involved in migration. Blood samples collected ≤3 years before diagnosis, a larger proportion of which likely represented cases who suffered from advanced EOC, displayed a somewhat larger variation and magnitude in expression, yet we did not observe statistically significant case-control differences in gene expression. Among genes previously linked to ovarian cancer, tests of *LIME1*, *GPR162*, *STAB1*, and *SKAP1* resulted in unadjusted p-values <0.05.

The prospective, population-based sampling was a major strength of this study, but the statistical power for explorative transcriptomics was limited. Including a greater number of samples or repeated measurements will allow closer investigation of whether transcript levels change during the course of EOC development.

## Supporting information

S1 Fig No separation of epithelial ovarian cancer cases and controls in hierarchical clustering of gene expression data. Cases shown in orange, controls in cyan. Dendrogram based on log_2_FC values of the 500 probes with lowest p-values in single-gene linear models of each case-control pair in all EOC.
(JPG)

S2 Fig No separation of epithelial ovarian cancer cases and controls in multidimensional scaling of gene expression data. Cases shown in orange, controls in cyan. Plot based on log_2_FC values of the 500 probes with lowest p-values in single-gene linear models of each case-control pair in all EOC.
(PNG)

S3 Fig Small differences in gene expression among the probes with the lowest p-values. Few log_2_FC values exceeded ±0.2. Volcano plot of log_2_FC values and p-values of the 100 probes with lowest p-values in single-gene linear models of all EOC.
(PNG)

S1 Table Mean estimated fractions of leukocyte populations in blood samples from all cases of epithelial ovarian cancer and controls. Based on deconvolution of gene expression values. P-value from a two-sided t-test of the mean difference.
(XLSX)

S2 Table The 100 probes with the lowest p-values in single-gene linear models (case-control) of gene expression in blood samples from cases of epithelial ovarian cancer (66 pairs). The presented p-values are not adjusted for multiple testing. All FDR q-values >0.96.
(XLSX)

S3 Table The 100 probes with the lowest p-values in single-gene linear models (case-control) of gene expression in blood samples from cases of metastatic epithelial ovarian cancer (56 pairs). The presented p-values are not adjusted for multiple testing. All FDR q-values >0.96.
(XLSX)

S4 Table The 100 probes with the lowest p-values in single-gene linear models (case-control) of gene expression in blood samples from cases of serous epithelial ovarian cancer (45 pairs). Almost all serous cases were metastatic. The presented p-values are not adjusted for multiple testing. All FDR q-values >0.96.
(XLSX)

S5 Table The 100 probes with the lowest p-values in single-gene linear models (case-control) of gene expression in blood samples collected ≤3 years before diagnosis (34 pairs). The presented p-values are not adjusted for multiple testing. All FDR q-values >0.96.
(XLSX)

S6 Table The 100 probes with the lowest p-values in single-gene linear models (case-control) of gene expression in blood samples collected >3 years before diagnosis (31 pairs). The presented p-values are not adjusted for multiple testing. All FDR q-values >0.96.
(XLSX)

S7 Table The 100 probes with the lowest p-values in single-gene linear models (case-control) of blood samples from all cases of epithelial ovarian cancer (EOC) (66 pairs) in models adjusted for leukocyte populations. Adjusted for estimated fractions of resting mast cells, plasma cells, neutrophils, monocytes, and CD8+ T cells (S1 Table). The presented p-values are not adjusted for multiple testing; all FDR q-values >0.96.
(XLSX)

S8 Table Background data for Fig 2. Enriched Gene Ontology (GO) categories for biological processes among the 100 probes with the lowest p-values in single-gene linear models (case-control) of blood samples from all cases of epithelial ovarian cancer (EOC), metastatic EOC (56 pairs), serous EOC (45 pairs, almost all were metastatic), and from blood samples collected ≤3 years or >3 years before diagnosis (34 and 31 pairs, respectively).
(XLSX)

S9 Table Summary of tests of genes identified in published functional genomics studies. Results from targeted tests of single genes identified in published studies investigating gene expression in peripheral whole blood or DNA methylation in circulating leukocytes from women with epithelial ovarian cancer.
(XLSX)
