# Supplementary figures and images for: The blood transcriptome prior to ovarian cancer diagnosis: A case-control study in the NOWAC postgenome cohort
Source: PLoS One. 2021 Aug 27;16(8):e0256442. doi: 10.1371/journal.pone.0256442 (PMC8396762; doi:10.1371/journal.pone.0256442)

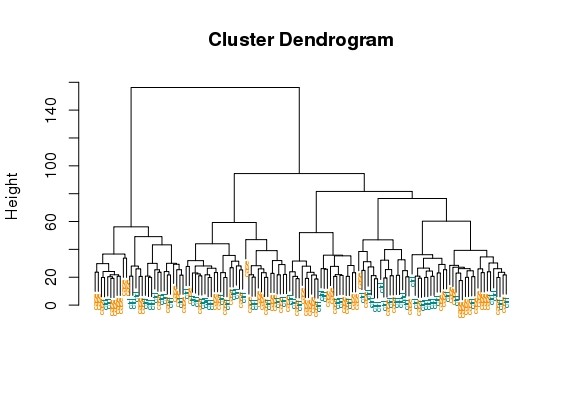

Supplement: S1 Fig — Cases shown in orange, controls in cyan. Dendrogram based on log2FC values of the 500 probes with lowest p-values in single-gene linear models of each case-control pair in all EOC. (JPG) [file pone.0256442.s001.jpg]

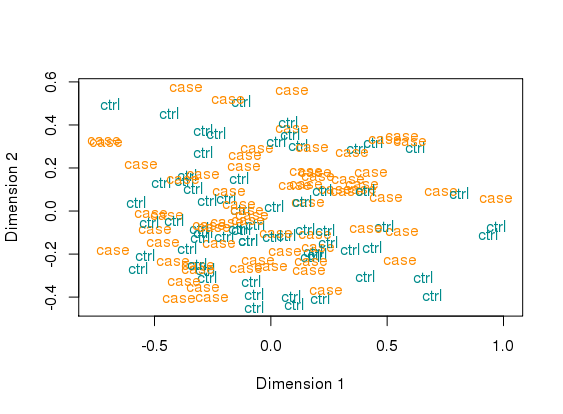

Supplement: S2 Fig — Cases shown in orange, controls in cyan. Plot based on log2FC values of the 500 probes with lowest p-values in single-gene linear models of each case-control pair in all EOC. (PNG) [file pone.0256442.s002.png]

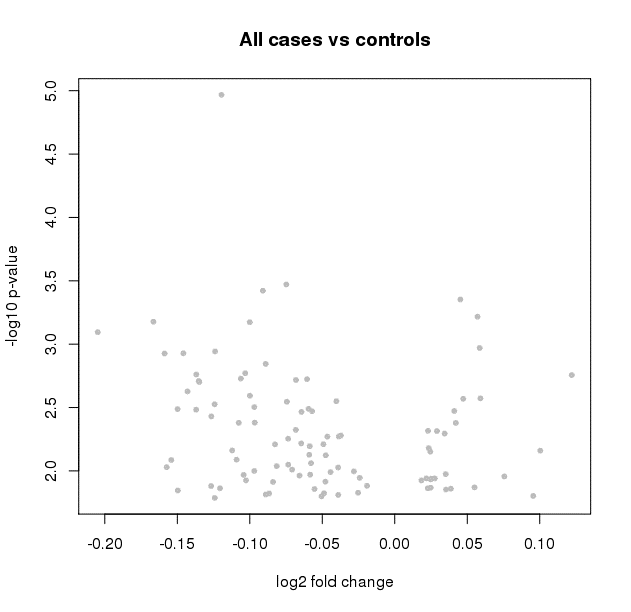

Supplement: S3 Fig — Few log2FC values exceeded ±0.2. Volcano plot of log2FC values and p-values of the 100 probes with lowest p-values in single-gene linear models of all EOC. (PNG) [file pone.0256442.s003.png]
